# Mismatch between femur and tibia coronal alignment in the knee joint: classification of five lower limb types according to femoral and tibial mechanical alignment

**DOI:** 10.1186/s12891-018-2335-9

**Published:** 2018-11-24

**Authors:** Yu-Hsien Lin, Feng-Shuo Chang, Kun-Hui Chen, Kui-Chou Huang, Kuo-Chih Su

**Affiliations:** 1 Department of Orthopedic Surgery, Taichung Veterans General Hospital, 1650, Taiwan Boulevard Sect. 4, Taichung City, 40705 Taiwan; 2 Department of Orthopedic Surgery, Asia University Hospital, 222, Fuxin Rd., Wufeng Dist, Taichung City, 41354 Taiwan; 3 Department of Occupational Therapy, Asia University, 500, Lioufeng Rd., Wufeng, Taichung City, 41354 Taiwan; 4 Department of Medical Research, Taichung Veterans General Hospital, 1650, Taiwan Boulevard Sect. 4, Taichung City, 40705 Taiwan; 5 RongHsing Research Center for Translational Medicine, National Chung Hsing University, 145, Xingda Rd., South Dist, Taichung City, 402 Taiwan; 6 Department of Biomedical Engineering, Hungkuang University, 1018, Sec. 6, Taiwan Boulevard, Shalu District, Taichung City, 43302 Taiwan; 7 Department of Orthopedics, Taichung Veterans General Hospital, Taichung, Taiwan; 8 Department of Biomedical Engineering, National Yang-Ming University, No. 155, Section 2, Linong St, Beitou District, Taipei City, Taiwan 112

**Keywords:** Normal knee, Coronal limb alignment, Mechanical alignment, Mismatch between femur and tibia, mLDFA, mMPTA

## Abstract

**Background:**

Reasons for dissatisfaction with total knee arthroplasty (TKA) include unequal flexion or extension gap, soft tissue imbalance, and patella maltracking, which often occur with mismatch between femoral and tibial coronal bony alignment in the knee joint or extremely varus or valgus alignment. However, lower limb coronal alignment classification is based only on hip–knee–ankle angle (HKAA), leading to oversight regarding a mismatch between femoral and tibial coronal alignment. We aimed to classify alignment of the lower limbs according to the mechanical alignment of the femur and tibia in a healthy population.

**Methods:**

All 214 normal triple films were reviewed retrospectively. HKAA, mechanical lateral distal femoral angle (mLDFA), mechanical medial proximal tibial angle (mMPTA), angle between the femoral anatomical axis and the mechanical axis (AA-MA), and knee alignment angle (KAA) were measured. Subjects were categorized into one of five types based on the mechanical alignment of femur and tibia.

**Results:**

Mean HKAA, mLDFA, and mMPTA of all subjects were 1.2°, 87.3°, and 85.8°, respectively. All subjects were classified into one of five types with significant differences (*p* < 0.001). About 61% of subjects showed neutral alignment, of which nearly 40% were type 2 (valgus of the femur and varus of the tibia with oblique joint line: mLDFA 85.0° ± 1.4°, mMPTA 85.1° ± 1.2°, TJLA 2.7° ± 2.4°) and 60% exhibited neutral alignment with a neutral femur and tibia (type 1). In varus and valgus types, mismatch between the mechanical angle of the femur and tibia was common. Varus alignment, including types 3 (varus of the tibia: mLDFA 88.0° ± 1.4°, mMPTA 83.5° ± 1.6°) and 4 (varus of both the tibia and femur: mLDFA 91.4° ± 1.4°, mMTPA 85.2° ± 2.0°), was found in 30% of subjects. Valgus alignment (type 5 valgus of femur: mLDFA 84.6° ± 1.6°, mMPTA 88.8° ± 2.0°) accounted for 8.9% of all subjects.

**Conclusions:**

Mismatch between mechanical alignment of the femur and tibia was common in varus and valgus alignment types. Joint line obliquity was also observed in 40% of the neutral alignment population. This classification provides a quick, simple interpretation of femoral and tibial coronal alignment, and more detailed guidance for preoperative planning for TKA than the traditional varus–neutral–valgus classification.

**Electronic supplementary material:**

The online version of this article (10.1186/s12891-018-2335-9) contains supplementary material, which is available to authorized users.

## Background

Restoration of neutral coronal alignment during total knee arthroplasty (TKA) plays a crucial role for the durability of the prosthesis [1, 2]. It is also considered to be an important parameter for predicting long-term aseptic wearing or loosening, according to previous literature [3, 4].

However, up to one-fifth of patients who underwent TKA are were dissatisfied [5–10]. Unequal flexion or extension gap, soft tissue imbalance, and patella maltracking might be the main reasons of dissatisfaction [11]. These problems frequently occurred during TKA on patients with a mismatch between femoral and tibial coronal alignment in the knee joint or with an extremely varus or valgus alignment. The imbalance could not always be corrected successfully by a soft tissue release technique unless comprehensive analysis of femoral and tibial coronal alignment was performed during preoperative planning.

In the aforementioned studies, lower limb coronal alignment was classified into varus, neutral, or valgus based on the hip–knee–ankle angle (HKAA) [12–18]. However, this classification does not describe the mismatch in the alignment of the tibia and femur. This study aimed to investigate and categorize the alignment of the knees in a healthy population according to natural femur and tibia mechanical alignments with a view to improving outcomes of TKA. The purposes of this study were (1) to analyze the lower limb coronal alignment of a healthy population, (2) to classify the alignment of the lower limbs according to the mechanical alignment of the femur and tibia, and (3) to discuss potential problems that could be encountered during TKA due to the mismatch of femoral and tibial coronal alignment for each type of lower limb, with the aim of improving outcomes of TKA.

## Methods

This retrospective, institutional review board-approved study was conducted by reviewing weight-bearing radiographs of the entire lower extremities (triple film) in our hospital that were taken in the outpatient department from January 2000 to December 2015 for any reason. In total, 2230 subjects aged 20–70 years were initially included for review. From these, healthy subjects were selected and included in this study. Unsuitable triple films were excluded according to the following criteria: previous complaint of knee or hip pain mentioned in the chart, osteoarthritis of the knee or hip on plain film (Kellgren–Lawrence classification grade 1 or above), lower limb trauma, deformity or surgery history, and rotation or poor image quality. All triple films were reviewed by two orthopedic research residents (the first and the second authors). Triple films of 1531 subjects with radiographic hip or knee osteoarthritis or subjective complaint of hip or knee pain, 352 subjects with a history of lower limb fractures, and 62 subjects with previous other lower limb surgeries (knee cruciate ligament reconstruction or other foot and ankle surgeries) were excluded, as well as 71 triple films with poor image quality. Finally, 214 qualified healthy subjects with normal lower limbs were included and were divided into two age groups (20–50 and 51–70 years).

In our hospital, full-leg antero-posterior radiographs are taken with patients standing barefoot, feet together, with fully extended knee and forward-oriented patellae to prevent rotation of the lower limbs [12]. The radiography tube is placed at a distance of 300 cm, and three cassettes are placed just behind the hips, knees, and feet. Digital stitching of these three radiographs is done for the final triple film.

**Fig. 1:**
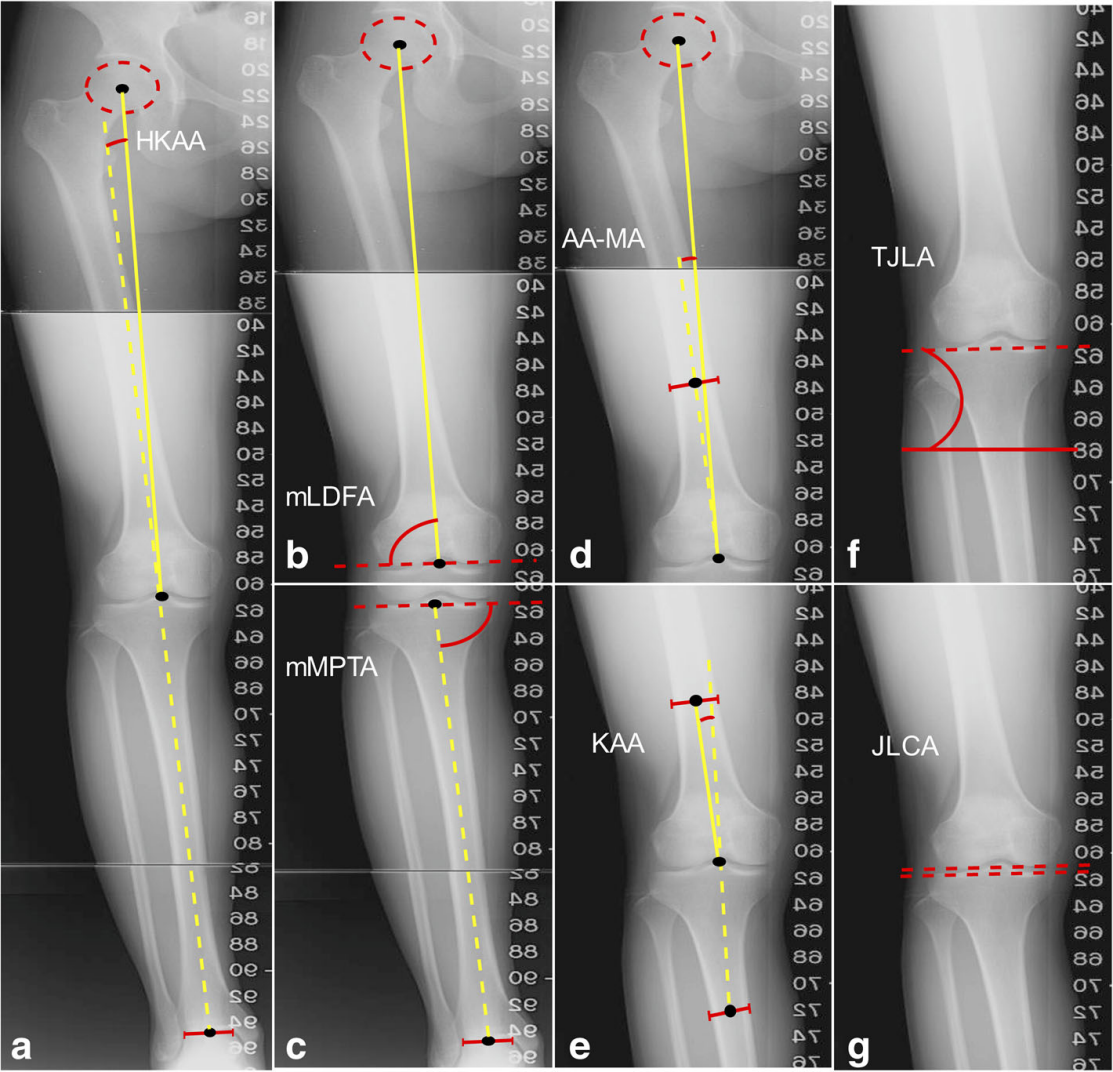
Measured Coronal Alignment Parameters. The five angles were defined as the following: **a** Hip–knee–ankle angle (HKAA): the angle between the mechanical axis of the femur and the tibia. The value of HKAA was defined as positive if varus alignment was found and as negative if valgus alignment was found. **b** Mechanical lateral distal femoral angle (mLDFA): the lateral angle between the mechanical axis of the femur and the distal femur joint line, which defined the connection of the lowest points of the medial and lateral femoral condyle. **c** Mechanical medial proximal tibial angle (mMPTA): the medial angle between the mechanical axis of the tibia and the proximal tibia joint line, which defined the connection of the lowest points of the medial and lateral tibial plateau. **d** Angle between the femoral anatomical axis and the mechanical axis (AA-MA): the angle between the mechanical axis and the anatomical axis of the femur. **e** Knee alignment angle (KAA): the angle between the anatomical axis of the femur and the anatomical axis of the tibia in the short film of the knee. The value of KAA was defined as positive if varus alignment was found and as negative if valgus alignment was found in the short film of the knee. **f** Tibial joint line angle (TJLA): the angle formed between the parallel line to the floor and the proximal tibia joint line. If both lines intersect with an angle on the lateral side of the leg, it is a medial open angle. If both lines intersect with an angle on the medial side of the leg, it is a lateral open angle. Positive values represent a lateral open angle and negative values represent a medial open angle. **g** Joint line convergence angle (JLCA): the angle between the knee joint lines of the distal femur and proximal tibia

Measured variables of lower limb coronal alignment [16–18] included HKAA (Fig. 1a), mechanical lateral distal femoral angle (mLDFA) (Fig. 1b), mechanical medial proximal tibial angle (mMPTA) (Fig. 1c), angle between the femoral anatomical axis and the mechanical axis (AA-MA) (Fig. 1d), knee alignment angle (KAA) (Fig. 1e), tibial joint line angle (TJLA) (Fig. 1f), and joint line convergence angle (JLCA) (Fig. 1g). Figure 1 illustrates and defines all angles. All measurements were performed independently by the same orthopedic research residents (the first and second authors) using the GeoGebra 5.0 software (International GeoGebra Institute, Austria, 2016). The average values of each variable according to age and sex were compared. The continuous variables were expressed as mean ± standard deviation (SD) and compared by one-sample t-test.

All subjects were categorized on the basis of the global limb alignment and mechanical alignment of the femur and tibia. Neutral alignment is HKAA within 3°, varus alignment is HKAA ≥3°; and valgus alignment is HKAA <− 3°. According to previous studies, the major contributors to valgus and varus knee are valgus of the distal femur [19] and varus of the proximal tibia [12], respectively. In this study, the mLDFA and mMPTA cut-off values were determined according to the mLDFA value in subjects with valgus alignment and the mMPTA value in subjects with varus alignment:

1. Varus of the tibia: The mean ± SD mMPTA of all subjects with varus alignment in this cohort was 84.03° ± 2.27°. Varus of the tibia was defined as mMPTA below 84.03° + 1 SD (84.03° + 2.27°). Therefore, the varus angle of the tibia was defined as mMPTA< 87° and the neutral angle of the tibia was defined as mMPTA≥87°.

2. Valgus of the femur: The average mLDFA value of all subjects with valgus alignment in this cohort was 84.6° ± 1.6°. Valgus of the femur was defined as mLDFA below 84.6° + 1 SD (84.6° + 1.6°). Therefore, the valgus angle of the femur was defined as mLDFA< 87° and the neutral angle of the femur was defined as 87° < mLDFA< 90°. An mLDFA value of ≥90° was defined as varus of the femur.

All subjects were classified into one of five types based on global limb alignment and combinations of these two variables (Table 1).

**Table 1:** Description of five types of lower limbs (classified according to mLDFA and mMPTA)

Group	Type/description	mLDFA^a^	mMPTA^a^
Neutral (− 3° ≤ HKAA < 3°)	1. Neutral alignment with normal joint obliquity (valgus of distal femur and varus of proximal tibia within 3°)	≥87°	≥87°
	2. Neutral alignment with high degree of joint line obliquity (valgus of distal femur and varus of proximal tibia above 3°)	< 87°	< 87°
Varus (HKAA ≥3°)	3. Genu varus with varus of the tibia	< 90°	< 87°
	4. Genu varus with varus of the tibia and femur	≥90°	< 87°
Valgus (HKAA <− 3°)	5. Genu valgus	< 87°	≥87°

^a^*mLDFA* mechanical lateral distal femoral angle, *mMPTA* mechanical medial proximal tibial angle

The incidence rate of each type was determined, and each variable (HKAA, mLDFA, mMPTA, AA-MA, KAA, TJLA, and JLCA) was analyzed between groups using one-way analysis of variance. Categorical variables were analyzed using Bonferroni post-hoc test or chi-square test. All statistical analyses were performed using SPSS version 22.0 (IBM Corp., Armonk, NY, USA). A *p*-value of < 0.05 was considered significant.

## Results

The average age of all 214 subjects was 41.3 years; 52% were male and 48% were female (Table 2 and Additional file 1). The alpha value of intra-class correlation coefficients of the two observers for HKAA, mLDFA, mMPTA, AA-MA, and KAA were 0.991, 0.912, 0.918, 0.964, and 0.797, respectively. The result indicated excellent inter-observer reliability.

**Table 2:** Analysis of lower limb coronal alignment based on sex and age (average age: 41.3 years)

Variables	All (*n* = 214)	Male (*n* = 112)	Female (*n* = 102)	*P*	20–50 y/o (*n* = 127)	51–70 y/o (*n* = 87)	*P*
HKAA(°) ^a^	1.2 ± 3.1	1.5 ± 3.0	0.8 ± 3.1	0.101	0.5 ± 2.6	2.2 ± 3.4	< 0.001
mLDFA(°) ^a^	87.3 ± 2.4	87.3 ± 2.3	87.2 ± 2.5	0.736	86.9 ± 2.2	87.8 ± 2.6	0.010
mMPTA(°) ^a^	85.8 ± 2.2	85.4 ± 2.2	86.2 ± 2.2	0.013	86.0 ± 2.1	85.5 ± 2.3	0.118
AA-MA(°) ^a^	4.7 ± 1.5	4.5 ± 1.4	4.9 ± 1.7	0.085	4.0 ± 1.1	5.8 ± 1.4	< 0.001
KAA(°) ^a^	−4.1 ± 2.3	−3.8 ± 2.3	−4.5 ± 2.3	0.028	−4.1 ± 2.1	−4.3 ± 2.6	0.529

^a^*HKAA* hip–knee–ankle angle, *mLDFA* mechanical lateral distal femoral angle, *mMPTA* mechanical medial proximal tibial angle, *AA-MA* angle between femoral anatomical axis and mechanical axis, *KAA* knee alignment angle

The mean HKAA of all study subjects was 1.2° (±3.1°). Although no statistical difference was observed, the mean HKAA value was higher in men (1.5° (±3.0°)) than in women (0.8° (±3.1°)) (Table 2). The mean mMPTA was lower in male subjects than in female subjects (*p* = 0.013), while the KAA was higher in male subjects than in female subjects (*p* = 0.028). The mean HKAA value was significantly higher in the older group than in the younger group (*p* < 0.01). Significantly more varus (*p* = 0.010) and more bowing (*p* < 0.001) of the femur were found in the older group. Although mMPTA had greater mean varus values in the older group, no statistical difference was observed.

**Fig. 2:**
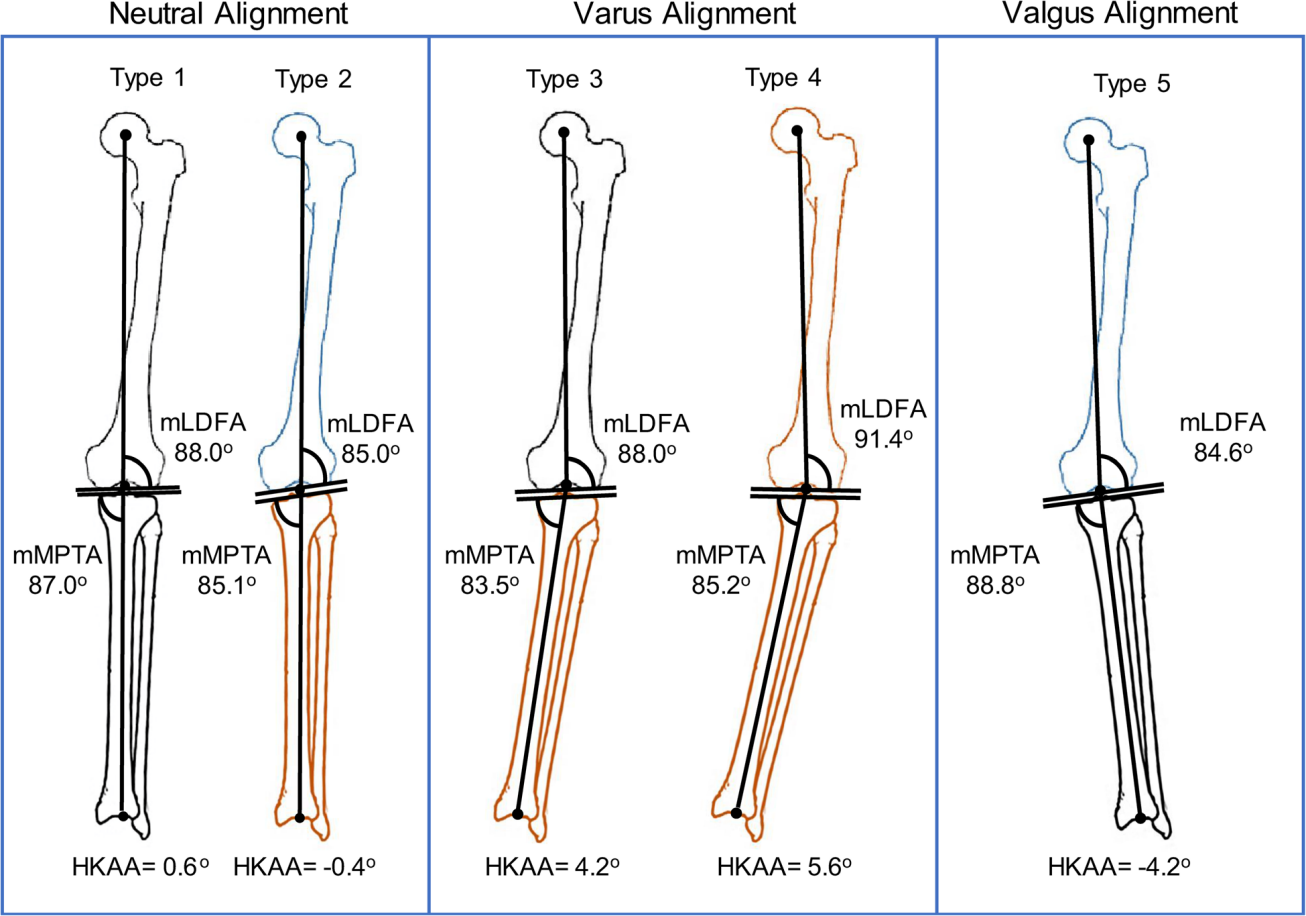
The Five Common Types of Normal Coronal Limb Alignment in a Taiwanese Population. The neutral alignment group consisted of two types (type 1 and type 2); the varus alignment group comprised 2 types (type 3, type 4); and the valgus alignment group consisted of one type (type 5). The black color indicates the femur or tibia in neutral alignment, the brown color indicates varus of the femur or tibia, and the blue color indicates valgus of the femur. HKAA, hip–knee–ankle angle; mLDFA, mechanical lateral distal femoral angle; mMPTA, mechanical medial proximal tibial angle

All subjects were successfully classified into one of five types of coronal alignment (Table 3 and Fig. 2). Approximately 61% of subjects showed neutral alignment, including type 1, which accounted for 36.9% of all subjects, and type 2, which accounted for 24.2% of all subjects. Type 2 (neutral alignment with the valgus angle of the femur, varus angle of the tibia, and an oblique joint line, with values statistically different from type 1) accounted for 40% of the neutral alignment types. The HKAA and KAA values of type 2 alignment were 1° more valgus than those of type 1. However, no statistical difference was observed.

**Table 3:** Classification of lower limb coronal alignment into five types based on mLDFA and mMPTA

Group	Knee types ^#^					
	Neutral		Varus		Valgus	
Variable	Type 1	Type 2	Type 3	Type 4	Type 5	*P*†
	Neutral	Oblique joint line	Varus of the tibia	Varus of both the tibia and femur	Valgus of the femur	
Number (N(total%))	79(36.9)	52(24.2)	43(20.0)	21(9.8)	19(8.9)	
Sex						0.155
Female (N(type%))	42(53.1)	27(51.9)	15(34.9)	7(33.3)	11(61.1)	
Age (Y)	38.2 ± 17.0	37.0 ± 18.0	47.0 ± 19.7	50.6 ± 18.9^b^	43.0 ± 18.2	0.005
Age group						0.001
20–50 year	56	36	17	7	11	
51–70 year	23	16	26	14	8	
HKAA(°)^*^	0.6 ± 1.6	−0.4 ± 1.4	4.2 ± 1.1^ab^	5.6 ± 2.3^abc^	−4.2 ± 0.9^abcd^	< 0.001
mLDFA(°)^*^	88.0 ± 1.3	85.0 ± 1.4^a^	88.0 ± 1.4^b^	91.4 ± 1.4^abc^	84.6 ± 1.6^acd^	< 0.001
mMPTA(°)^*^	87.0 ± 1.5	85.1 ± 1.2^a^	83.5 ± 1.6^ab^	85.2 ± 2.0^ac^	88.8 ± 2.0^abcd^	< 0.001
AA-MA(°)^*^	4.4 ± 1.1	4.2 ± 1.2	5.4 ± 1.6^ab^	6.0 ± 2.1^ab^	4.0 ± 1.7^cd^	< 0.001
KAA(°)^*^	− 4.2 ± 1.5	−5.2 ± 1.5^a^	− 2.2 ± 1.7^ab^	−1.8 ± 1.7^ab^	−7.8 ± 2.4^abcd^	< 0.001
TJLA(°) ^*^	0.0 ± 1.6	2.7 ± 2.4^a^	0.9 ± 2.1^ab^	−0.9 ± 2.2^bc^	2.0 ± 3.3^ad^	< 0.001
JLCA(°) ^*^	−0.4 ± 0.7	−0.2 ± 0.7	− 0.2 ± 1.1	−0.2 ± 1.2	− 0.5 ± 0.5	0.510

^*^HKAA hip–knee–ankle angle, *mLDFA* mechanical lateral distal femoral angle, *mMPTA* mechanical medial proximal tibial angle, *AA-MA* angle between femoral anatomical axis and mechanical axis, *KAA* knee alignment angle, *TJLA* tibial joint line angle, *JLCA* joint line convergence angle

^#^ Type 1: valgus of the distal femur and varus of the proximal tibia within 3°; type 2: valgus of the distal femur and varus of the proximal tibia above 3° (neutral with knee joint line obliquity); type 3: genu varus with varus of the tibia; type 4: genu varus with both varus of the tibia and femur; type 5: genu valgus

^a^*P* < 0.05 vs. type 1;^b^*P* < 0.05 vs. type 2;^c^*P* < 0.05 vs. type 3;^d^*P* < 0.05 vs. type 4

^†^denotes one-way analysis of variance (ANOVA) for continuous variables with Bonferroni *post-hoc* test or chi-square test for categorical variables

Varus alignment types accounted for 30% of the total subjects. Most of them were classified as type 3, which comprised 67% of all varus alignment types. The average HKAA, mLDFA, and mMPTA were 4.2° ± 1.1°, 88.0° ± 1.4° (similar to type 1 and statistically different from type 2), and 83.5° ± 1.6° (more tibial varus than types 1 and 2), respectively. The joint line was nearly horizontal to the floor (TJLA 0.9° ± 2.1°) despite the value being statistically different from type 1. This result indicates that in type 3 varus knees, the femur was in neutral alignment, and the major contributor to varus was varus of the tibia. Type 4 accounted for 33% of varus alignment types, with a greater varus HKAA (5.6° ± 2.3°) that was statistically different from type 3 varus knee. In this type, varus of both the femur and the tibia was present (mLDFA 91.4° ± 1.4°, statistically different from types 1, 2, and 3; mMPTA 85.2° ± 2.0°, statistically different from types 1 and 3, and similar to type 2). Joint line orientation was also nearly horizontal to the floor in type 4, with mild medial opening (TJLA -0.9° ± 2.2°). Despite less varus alignment of the tibia than was observed in type 3, type 4 was the type found to show varus of both the femur and tibia. The average subject age in this type was also higher than that of other types. In the varus group, the femur showed significantly more bowing, with an AA-MA of 5.4° ± 1.6° and 6.0° ± 2.1° in types 3 and 4, respectively, and a more varus KAA of − 2.2 ± 1.7 and − 1.8° ± 1.7° in types 3 and 4, respectively, than the neutral types.

Type 5 valgus alignment accounted for 9% of all subjects, and the average HKAA was − 4.2° ± 0.9°, with mLDFA of 84.6° ± 1.6° (statistically different from types 1, 3, 4, but similar to type 2) and neutral mMPTA of 88.8° ± 2.0°. This indicates that the major contributor to valgus alignment was valgus of the femur. The joint line of type 5 was more oblique than that of types 1 and 4, with a statistical difference. The JLCA of all types was nearly 0°, with no statistical difference between types.

The incidence rates of each type of knee in the two age groups were analyzed (Table 3). In the younger group, neutral alignment types were identified in 72% of the subjects. However, in the older group, varus alignment types were identified in 66% of subjects. Of interest, in both age groups, type 2 accounted for approximately 40% of neutral alignment subjects. Neutral alignment with oblique joint line was very common in the neutral alignment population, regardless of age.

## Discussion

To ensure greater longevity of the knee prosthesis in TKA, restoring neutral coronal limb alignment is necessary [20, 21]. The natural knee with neutral alignment has an HKAA of nearly 0°, with mLDFA and mMPTA of approximately 87° ± 3° [12–18]. Thus, when the distal femur and proximal tibia are cut perpendicular to the mechanical axis of the femur and tibia, external rotation of the femur by approximately 3° balances the flexion gap [22]. However, up to one-fifth of patients who received TKA have reported being dissatisfied with the surgical outcome [5–10]. Unequal flexion or extension gap, soft tissue imbalance, and patella maltracking may lead to dissatisfaction [11]. In previous studies, 32% of males and 17.2% of females in a Western population [12] and 20.34% of Korean females [17] had constitutional varus (HKAA > 3°). Among these populations, “correction” of the lower limb coronal alignment to neutral in these populations might induce iatrogenic soft tissue imbalance. In another study, a > 5° varus of the tibia was observed in the coronal alignment of the lower extremities among Chinese adults [18]. In these subjects, more medial–lateral soft tissue imbalance might be encountered during TKA. Thus, analysis of mechanical alignment of the femur and tibia during preoperative planning might help clinicians to manage soft tissue imbalance.

In the aforementioned studies, lower limb coronal alignment was classified into varus, neutral, or valgus based on HKAA [12–18]. However, this classification does not describe the mismatch in the alignment of the tibia and femur. It is of great importance to have a comprehensive understanding of the bony alignment of both the femur and tibia separately and their relation to lower limb coronal alignment in the preoperative planning for TKA. The three major contributions of the present study are (1) the successful classification of the alignment of the lower extremities into five types, with statistically significant differences, based on mLDFA and mMPTA; (2) the finding that mismatch between mechanical alignment of the femur and tibia was common in varus and valgus alignment types; and (3) the finding that joint line obliquity was observed in 40% of the neutral alignment population.

Types 1 and 2 were both neutral in global knee alignment, but were significantly different in femoral and tibial mechanical alignment. Due to the valgus angle of the femur and varus angle of the tibia, an oblique joint line was observed in type 2 alignment. This may cause direct biomechanical consequences of joint loading and shear stress [23]. However, the direct consequences of weight transmission in vivo and the relationship to knee joint degeneration should be investigated in a future study. To the best of our knowledge, this study is the first to report this type of alignment. In a patient with knee osteoarthritis with type 1 and 2 neutral alignment before osteoarthritis, a balanced extension gap might be achieved by performing a bony cut perpendicular to the mechanical axis during TKA.

In this study, 39% of the subjects had either the varus or valgus alignment type, and a mismatch between natural mLDFA and mMPTA was observed. A mismatch between the femur and tibia might cause iatrogenic soft tissue imbalance if the bony cut performed during TKA is done perpendicular to the mechanical axis of the femur and tibia. However, correcting this imbalance using the soft tissue balancing technique is not always possible. Type 3 varus alignment accounted for 20% of all subjects with mLDFA of 88.0° ± 1.4° and mMPTA of 83.5° ± 1.6°. The major contributor to the deformity in type 3 was the varus angle of the tibia, explaining why osteotomy of varus osteoarthritis is usually performed in the proximal tibia [24]. Moreover, the horizontal joint line orientation may be preserved after osteotomy. If the mechanically aligned measured resection technique is used for TKA in a patient with knee osteoarthritis with type 3 alignment before osteoarthritis, bone resection might cause 4.5° tightness at the medial side of the coronal plane, which would require medial soft tissue release. However, if the mechanically aligned gap balancing technique is used with the tibial bone cut first, the varus femur component in the coronal plane might elevate the joint line, and a high degree of femur component external rotation might change the patella tracking in the transverse plane [25]. Thus, balancing of the soft tissue may become more difficult. Residual varus alignment in flexion may also occur due to excessive external rotation of the femoral component [22].

The second common type of varus alignment was type 4 with mLDFA of 91.4° ± 1.4° and mMTPA of 85.2° ± 2.0°. In this type, the coronal lower limb alignment was more varus than in all other types (5.6° ± 2.3°, *p* < 0.05). In patients with knee osteoarthritis with type 4 varus alignment before osteoarthritis, 6.2° tightness in the extension gap might need to be corrected by soft tissue release at the medial side after a perpendicular bony cut to the mechanical axis during TKA. An extensive medial side soft tissue release needs to be done to obtain a balanced extension gap. However, overzealous medial side soft tissue release in extension causes flexion gap opening in the medial side in the measured resection technique [26]. This causes flexion instability of the knee joint, and an even more constrained TKA prosthesis may be needed. Nagamine et al. [27] mention that anatomic variation should be considered during TKA in these types of patients. The joint line orientation of these two types of varus alignment was nearly horizontal to the floor, which is similar to type 1 neutral alignment. This result is consistent with the report of Victor et al. [23]. However, in comparison to type 3 varus knee, type 4 has a more medial opening joint line orientation, which may be caused by varus of the femur and might result in a greater medial opening during the osteoarthritis process (TJLA: − 1.9° ± 3.5° in a previously reported osteoarthritis cohort [23] versus TJLA: − 0.9° ± 2.2° in type 4 alignment in the present study). Therefore, a future prospective study of these patients should be conducted to determine whether individuals with type 4 alignment have a higher risk of osteoarthritis. It is also important to note that types 3 and 4 both show more severe bowing of the femur than is observed in the neutral group. Thus, caution should be observed while using an intramedullary guide for distal femur resection.

Type 5 consisted of about 9% of all subjects in this study, with an average HKAA of − 4.2° ± 0.9°, mLDFA, 84.6° ± 1.6°; and mMPTA, 88.8° ± 2.0°. In valgus alignment, the major contributor is the femur, and the tibia is the most neutral among all types. Due to valgus of the femur and an inability to bring the feet together, an oblique joint line was also observed in type 5 valgus alignment, which is comparable to previously reported results [23]. Therefore, distal femur osteotomy is performed in patients with valgus osteoarthritis [28], and the joint line in these patients may also be corrected to be horizontal to the floor after the osteotomy. In patients with knee osteoarthritis with type 5 valgus alignment before osteoarthritis, perpendicular bony cut to the mechanical axis during TKA causes approximately 4.2° of extension gap tightness in the lateral side. Therefore, lateral release such as an ilio-tibial band release is often needed to achieve balanced soft tissue tension.

Differences were observed between the younger and older groups in this study (Tables 2 and 3). The mean HKAA value was higher in the older group than in the younger group (2.2° ± 3.4° and 0.5° ± 2.6°, respectively, *p* < 0.001). Greater varus (mLDFA) and bowing (AA-MA) of the femur in the older group were also observed. Moreover, the prevalence rates of types 3 and 4 were higher in the older group than in the younger group. This finding might be explained by Hueter–Volkmann’s law, which states that suppression of physis growth occurs under compression force and stimulation of physis growth occurs when loading is reduced [12, 29, 30]. The difference in daily habits and lifestyle between the two generations (older Taiwanese populations tend to have engaged in more manual labor and often squatted while undertaking agricultural work) might have contributed to secondary varus alignment, due to accelerated growth in the lateral physis and delayed growth in the medial physis.

Finally, we compared our results to previous findings in the literature (Table 4). The analysis of alignment of the lower limbs in an Asian population in this study revealed a similar mean value of HKAA to values reported in the literature. In the present study, we found a mean HKAA of 1.2° (±3.1°), which was similar to previous study results of 1.3° (±2.3°) among Belgians [12], 1.5° (±2.0°) among Western males [16], 1.5° (±2.9°) among Iranians [14], 1.4° (±2.0°) among Korean females [17], and 2.2° (±2.7°) among Chinese [18]. The presence of a slight varus deviation from neutral mechanical knee alignment was common, regardless of race. The mean HKAA of men (1.5° (±3.0°)) in the present study was more varus than in women (0.8° (±3.1°)), which was also similar to previously reported values by Bellemans et al. [12] and Jabalameli et al. [14]. Despite finding a mean HKAA consistent with previously reported values, the present study is the first to report a marked mismatch between natural femur and tibia mechanical alignment in varus and valgus knees and joint line obliquity in two-thirds of neutral knees.

**Table 4:** Comparison of lower limb alignment values reported from previous studies

	Bellemans et al. (Belgium)			Moreland et al. (Caucasian)	Jabalameli et al. (Iran)			Song et al. (Korea)	Tang et al. (Hong Kong)		Current study	
	All	Male	Female	Male	All	Male	Female	Female	Male	Female	Male	Female
HKAA(°)	1.3 ± 2.3	1.9 ± 2.4	0.8 ± 2.1	1.5 ± 2.0	1.5 ± 2.9	3.0 ± 3.1	0.7 ± 2.7	1.4 ± 2.0	2.2 ± 2.7	2.2 ± 2.5	1.5 ± 3.0	0.8 ± 3.1
mLDFA(°)	87.9 ± 1.7	87.9 ± 1.7	87.9 ± 1.8	88.5 ± 2.0	88.9 ± 3.0	89.2 ± 3.3	88.5 ± 2.7	87.8 ± 1.7	87.3 ± 2.7	86.8 ± 2.5	87.3 ± 2.3	87.2 ± 2.5
mMPTA(°)	87.0 ± 2.1	86.5 ± 2.2	87.6 ± 1.8	87.0 ± 1.6	87.2 ± 2.0	86.4 ± 1.7	88.0 ± 2.0	86.8 ± 1.6	85.1 ± 2.3	84.6 ± 2.5	85.4 ± 2.2	86.2 ± 2.2
AA-MA(°)	4.5 ± 0.6	4.5 ± 0.6	4.4 ± 0.5	5.8 ± 0.7	5.7 ± 1.2	5.7 ± 1.0	5.7 ± 1.4	6.0 ± 0.7	5.6 ± 0.8	5.7 ± 1.0	4.5 ± 1.4	4.9 ± 1.7
KAA(°)					−3.9 ± 3.4	−2.8 ± 3.7	−5.2 ± 2.8				−3.8 ± 2.3	−4.5 ± 2.3
Varus(%)	24.6%	32%	17.2%					20.3%			37.5%	21.6%

*HKAA* hip–knee–ankle angle, *mLDFA* mechanical lateral distal femoral angle, *mMPTA* mechanical medial proximal tibial angle, *AA-MA* angle between the femoral anatomical axis and the mechanical axis, *KAA* knee alignment angle

Despite the novelty of this study, a few limitations should be considered. First, all the subjects were selected, reviewed, and analyzed retrospectively by two orthopedic research residents. Some selection bias may exist, such as the inadvertent selection of slightly abnormal triple film despite the exclusion of all abnormal triple films. However, the average HKAA in this study showed results similar to those reported in previous studies, indicating that the influence of selection bias was likely minimal. The second limitation was that full-leg standing coronal plain film was used for measurements in this study. Although this method has been confirmed to have excellent intra- and inter-observer reliability in previous studies, rotation of the extremities might have influenced the accuracy of the measurements [31–36]. However, in this study, the forward-oriented patella might have minimized the effect of rotation in most subjects, as in many previous studies of lower leg alignment [16, 34, 36–38]. Furthermore, the role of lower limb morphology in the transverse and sagittal plane is also crucial in the evaluation of lower limb alignment and preoperative planning, but such information could not be obtained by plain film. Computed tomography imaging of the lower limbs is another choice to avoid the influence of limb rotation and obtain lower limb morphology in the transverse and sagittal planes [39]. However, higher exposure to radiation exposure is always a concern. The third limitation was that four of the 19 valgus subjects had valgus alignment with mMPTA ≥90; these accounted for a very small proportion of our study population. This study aimed to propose a quick way to interpret lower limb axial alignment for common types of lower limb alignment. Therefore, this study did not classify these patients as type 6 but included them in type 5 valgus alignment. However, these account for 21% of all cases of valgus alignment. Thus, this population would be investigated in a future larger scale study. The last limitation of this study was that the correlation of femur and tibia mechanical alignment between healthy populations and patients with osteoarthritis was unknown despite the fact that bony destruction is only present in advanced osteoarthritis. The findings presented herein might be useful for quick and simple interpretation of each type of alignment and may be used in the first step of TKA preoperative planning, except in cases of severe knee osteoarthritis with secondary bony destruction. However, the results should also be interpreted cautiously, and treatment should be adjusted according to each patient’s individual condition.

## Conclusion

In conclusion, this study successfully classified the alignment of the lower extremities into five types, based on mLDFA and mMPTA. Mismatch between mechanical alignment of the femur and tibia was common in varus and valgus alignment types. Joint line obliquity was also observed in 40% of the neutral alignment population. This classification might provide a quick and simple interpretation of femoral and tibial coronal alignment and provide guidance for preoperative planning for TKA that is more detailed than the traditional varus–neutral–valgus classification, thereby increasing the likelihood of obtaining an optimal balance between bony alignment and soft tissue.

## Additional file

Additional file 1: Raw data. (XLSX 22 kb)

## Abbreviations

AA-MA: angle between the femoral anatomical axis and the mechanical axis
HKAA: hip–knee–ankle angle
JLCA: joint line convergence angle
KAA: knee alignment angle
mLDFA: mechanical lateral distal femoral angle
mMPTA: mechanical medial proximal tibial angle
SD: standard deviation
TJLA: tibial joint line angle
TKA: total knee arthroplasty
